# Comparative effectiveness of low-dose-rate brachytherapy with or without external beam radiotherapy in favorable and unfavorable intermediate-risk prostate cancer

**DOI:** 10.1038/s41598-022-15028-6

**Published:** 2022-06-30

**Authors:** Hideyasu Tsumura, Nobumichi Tanaka, Tomohiko Oguchi, Takuya Owari, Yasushi Nakai, Isao Asakawa, Kazuyoshi Iijima, Haruaki Kato, Iwao Hashida, Ken-ichi Tabata, Takefumi Satoh, Hiromichi Ishiyama

**Affiliations:** 1 Department of Urology, Kitasato University School of Medicine, 1-15-1 Kitasato Minami-ku, Sagamihara, Kanagawa 252-0374 Japan; 2 Department of Urology, Nara Medical University, Kashihara, Japan; 3 Department of Urology, Nagano Municipal Hospital, Nagano, Japan; 4 Department of Radiation Oncology, Nara Medical University, Kashihara, Japan; 5 Department of Radiation Therapy, Nagano Municipal Hospital, Nagano, Japan; 6 Department of Radiation Oncology, Kitasato University School of Medicine, Sagamihara, Japan

**Keywords:** Cancer, Urology

## Abstract

We compared clinical outcomes associated with seed brachytherapy (SEED-BT) alone and SEED-BT plus external-beam radiotherapy (EBRT) for intermediate-risk prostate cancer using propensity score-matched analysis. From 2006 to 2011, 993 patients diagnosed with intermediate-risk were treated with either SEED-BT alone (*n* = 775) or SEED-BT plus EBRT (*n* = 158) at 3 tertiary hospitals. In the propensity score-matched analysis (102 pairs), median follow-up was 95 months (range 18–153 months). The 8-year biochemical recurrence-free rate (bRFR) was significantly better with SEED-BT alone than with combined radiotherapy (93.3% vs. 88.4%; HR 0.396; 95% CI 0.158–0.991). Grade 2 or greater late genitourinary toxicities were significantly fewer with SEED-BT alone than with combined radiotherapy (21.0% vs. 33.2%; HR 0.521; 95% CI 0.308–0.881). Similarly, grade 2 or greater late gastrointestinal toxicities were significantly fewer with SEED-BT alone (0% vs. 12.2%; HR 0.125; 95% CI 0.040–0.390). For the unfavorable intermediate-risk subgroups, SEED-BT alone yielded a significantly better bRFR than the combined radiotherapy (HR 0.325; 95% CI 0.115–0.915). SEED-BT alone might be a better disease-management plan than SEED-BT plus EBRT for intermediate-risk prostate cancer regardless of favorable and unfavorable characteristics.

## Introduction

Permanent seed brachytherapy (SEED-BT) has taken a place alongside external-beam radiotherapy (EBRT) and radical prostatectomy as one of the definitive therapeutic options for treating intermediate-risk prostate cancer. The most appealing reasons for selecting this treatment are favorable disease control rates and acceptable side effect profiles^1–6^. Prostate brachytherapy for patients with intermediate-risk disease has conventionally used a combination of SEED-BT and EBRT rather than SEED-BT alone^7^, especially if unfavorable factors such as a higher Gleason score and higher-volume disease are present. Although the ASCENDE-RT (Androgen Suppression Combined with Elective Nodal and Dose Escalated Radiation Therapy ) randomized trial demonstrated that a combination of SEED-BT and EBRT improved biochemical control over dose-escalated EBRT (78 Gy) alone in disease classified as high- and intermediate-risk^8^, no current consensus has been reached about whether the combination of brachytherapy and EBRT provides a better clinical outcome over brachytherapy alone in intermediate-risk prostate cancer. Some studies reported a biochemical control advantage for combined therapy^9^. Others showed no biochemical control improvement for combined radiotherapy compared with SEED-BT alone^10^.

In the present study, we used a propensity-score-matching (PSM) analysis to compare clinical outcomes and toxicities associated with SEED-BT alone and SEED-BT plus EBRT for the treatment of intermediate-risk prostate cancer. PSM minimizes the imbalance of patient characteristics when two treatment groups are being compared. Our question was whether additional treatment with EBRT, compared with SEED-BT alone, results in superior oncologic outcomes in intermediate-risk prostate cancer in the era of technically advanced SEED-BT.

## Methods

We identified patients with intermediate-risk prostate cancer (prostate-specific antigen [PSA] 10–20 ng/mL or stage T2b–c disease or Gleason score 7) who underwent SEED-BT alone or SEED-BT plus EBRT in 3 tertiary hospitals between January 2006 and December 2011. Data from these patients’ medical records were extracted into a compatible-format database. The original data from the medical records was used in the present study. The biopsy slides and radiographic images were not reviewed as part of this study. For the purpose of analysis, the U.S. National Comprehensive Cancer Network 2019 guidelines (version 4) were used to categorize their disease into favorable and unfavorable intermediate-risk. Patients with no evidence of biochemical recurrence at less than 2 years of follow-up were excluded. Patients who died from any cause or those who developed biochemical recurrence within 2 years after radiotherapy were included. To minimize the impact of hormonal therapy on time to biochemical recurrence, patients who received more than 12 months of neoadjuvant hormonal therapy (NHT) were excluded from the study. Patients who received any kind of adjuvant hormonal therapy were also excluded^11^. Tc99m bone scintigraphy was routinely performed to detect bone metastasis for pretreatment evaluation. Either pelvic/abdominal computed tomography or pelvic magnetic resonance image were used to detect the nodal and distant metastasis. Digital rectal examination, transrectal ultrasonography, and magnetic resonance image were used for the local evaluation of the prostate. Pelvic magnetic resonance image was routinely performed unless there were contraindications for performing the examination.

### Ethics approval and consent to participate

The database was closed for analysis in December 2018. This retrospective study was approved by Kitasato University Medical Ethics Organization (B18-205). All methods were carried out in accordance with relevant guidelines and regulations. Informed consent was obtained in the form of opt-out in the web-site. Those who rejected were excluded.

### Treatment protocol at each institution

Some patients receiving SEED-BT at institution A in 2006 were treated using preplanning methods. Most other patients at the 3 institutions were treated using an intraoperative planning method with modified peripheral loading techniques using a Mick applicator^12,13^. The therapeutic planning and post-implant dosimetric evaluation were performed using the Interplant planning system (CMS, St. Louis, MO) or Variseed (Varian, Palo Alto, CA). ^125^I was used for all patients. Either Oncoseed 6711 (GE Healthcare, Arlington Heights, IL) or STM 1251 (BD, Tempe, AZ) was used for SEED-BT. The doses were defined using the TG-43 criteria^14^. At 1 month after treatment with SEED-BT alone, a computed tomography-based dosimetric analysis was performed to calculate the D90, V100, and V150 results. Prostate D90 is the minimum dose to 90% of the prostate gland at 1 month. Prostate V100 and V150 are the percentages of the prostate gland volume respectively receiving 100% and 150% of the prescribed dose at 1 month. These treatment protocols were used at each institution:

#### Institution A

Patients were treated with a combination of SEED-BT and EBRT if at least 1 of the following factors was present: PSA exceeding 10 ng/mL but less than or equal to 20 ng/mL, Gleason grade group 3, a positive biopsy core rate of 50% or greater, or stage T2b–c disease. Other patients classified as intermediate-risk were treated with SEED-BT alone. From January 2006 to April 2007, patients receiving SEED-BT alone were treated at a prescribed dose of 145 Gy; from May 2007 to December 2011, they were treated at a prescribed dose of 160 Gy. Patients receiving combined SEED-BT and EBRT were treated at a prescribed SEED-BT dose of 110 Gy. The EBRT target was determined 1 month after SEED-BT, and patients received 45 Gy (in 25 fractions of 1.8 Gy each) using 10 MV of photon energy (3-dimensional conformal radiotherapy). The clinical target volume (CTV) for EBRT was defined as the prostate. The planning target volume (PTV) for EBRT was created by adding an 8 mm margin surrounding the CTV, except posteriorly, where the margin was limited to 3 mm. The CTV for SEED-BT included the entire prostate. No PTV was created in SEED-BT.

#### Institution B

Patients were treated with a combination of SEED-BT and EBRT if at least 1 of the following factors was present: PSA of 10 ng/mL to 20 ng/mL or less, or Gleason grade group 3. Other patients classified as intermediate-risk were treated with SEED-BT alone. Patients receiving SEED-BT alone were treated at a prescribed dose of 160 Gy. Patients receiving combined SEED-BT and EBRT were treated at a prescribed SEED-BT dose of 100 Gy. EBRT was completed 2 weeks before SEED-BT, and those patients received 46 Gy (in 23 fractions of 2 Gy each) using 10 MV of photon energy (3-dimensional conformal radiotherapy). The CTV for EBRT was defined as the prostate and one third of the proximal seminal vesicle. The PTV for EBRT was created by adding a 10-mm margin surrounding the CTV, except posteriorly, where the margin was limited to 6 mm. The CTV for SEED-BT was the prostate as identified under transrectal ultrasound guidance. The PTV for SEED-BT was generated using a 3 mm expansion of the CTV, except posteriorly, where no margin was applied.

#### Institution C

All patients classed as intermediate-risk were candidates for treatment with SEED-BT alone. Patients receiving SEED-BT alone were treated at a prescribed dose of 145 Gy. Non patients were treated with a combination of SEED-BT and EBRT. The CTV for SEED-BT included the entire prostate. No PTV was created in SEED-BT.

### NHT

None of the 3 institutions had a fixed protocol for NHT. The major reason to use NHT was to reduce the size of the prostate for gland volumes exceeding 50 cm^3^, which can make seed implantation technically more difficult. Other patients received NHT depending on the strategy of the treating physician. Some patients received a combination of an oral nonsteroidal anti-androgen agent with injection of a gonadotropin-releasing hormone agonist; others received either nonsteroidal anti-androgen agents alone or injection of a gonadotropin-releasing hormone agonist alone. Either flutamide (250–375 mg daily) or bicalutamide (80 mg daily) was used as the nonsteroidal anti-androgen agent. Either goserelin (3.6 mg monthly or 10.8 mg every 3 months) or leuprorelin (3.75 mg monthly or 11.25 mg every 3 months) was given as gonadotropin-releasing hormone agonist therapy.

### Outcome measures

The primary outcome was the biochemical recurrence-free rate (bRFR); secondary outcomes were the salvage hormonal-therapy-free rate, the metastasis-free survival rate, and the incidence of genitourinary (GU) and gastrointestinal (GI) toxicity. Day 0 was the day on which patients were treated with either SEED-BT alone or SEED-BT followed by EBRT. When EBRT started before SEED-BT, Day 0 was the first day of EBRT. Biochemical recurrence was defined using the Phoenix definition (PSA nadir plus 2 ng/mL) after 24 months from radiotherapy^15^. Regional lymph node metastasis and distant metastasis were both classified simply as metastasis in the analysis for metastasis-free survival. Dose comparisons between SEED-BT alone and SEED-BT plus EBRT used biological effective dose (BED) equations with an α/β ratio of 2, following Stock et al*.*^16^. Toxicity was defined as any event developing after the initiation of radiotherapy and was scored using the *Common Terminology Criteria for Adverse Events,* version 4. Toxicity occurring within 3 months after initiation of radiotherapy was defined as acute toxicity. Toxicity occurring more than 3 months after initiation of radiotherapy was defined as late toxicity.

### Statistical analyses

Patient characteristics in the SEED-BT alone and SEED-BT plus EBRT groups were compared using the chi-square test for categorical variables and the Wilcoxon rank-sum test for continuous variables. To correct for potential bias in treatment selection, we used the teffects psmatch function (StataCorp, College Station, TX [2013]) to conduct a propensity-score-matched analysis^17,18^. We used the same function to estimate the average effect of SEED-BT plus EBRT treatment on biochemical recurrence. To calculate the propensity score for treatment with SEED-BT plus EBRT, the variables used were age, PSA at diagnosis, Gleason grade groups, clinical stage, positive biopsy core rate, and administration of NHT. The presence or absence of NHT was used in calculating the propensity score, but the duration or kinds of NHT were not considered. A propensity score analysis then performed 1:1 nearest-neighbor matching.

After pairs had been matched, outcome measures were compared. The Kaplan–Meier method was used to estimate survival rates, and differences were assessed using the log-rank statistic. The Kaplan–Meier method was also used to estimate the cumulative incidences of grade 2 and greater and 3 and greater late GU and GI toxicities. Mantel–Haenszel hazard ratios were calculated for those outcomes.

Differences were regarded as statistically significant at *p* < 0.05. All reported *p* values are two-sided. All analyses were performed in the Stata (version 15: StataCorp) and GraphPad Prism (version 8: GraphPad Software, La Jolla, CA) software applications.

## Results

**Figure 1:**
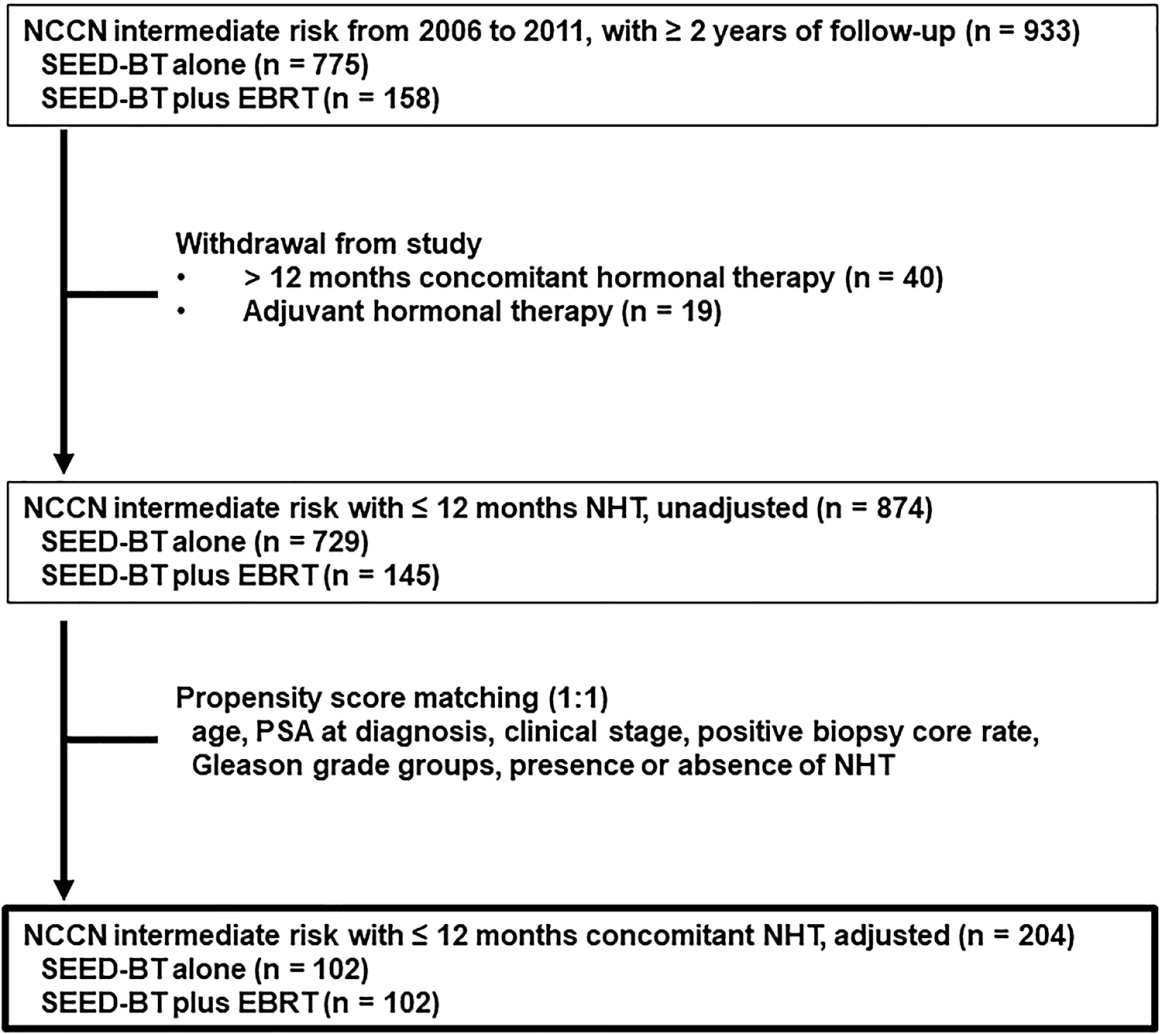
Patient selection for the study. EBRT = external-beam radiotherapy; NCCN = U.S. National Comprehensive Cancer Network guidelines (2019, version 4); NHT = neoadjuvant hormonal therapy; PSA = prostate-specific antigen; SEED-BT = seed brachytherapy.

Figure 1 shows the patient selection process. Between January 2006 and December 2011, 933 patients with intermediate-risk prostate cancer and more than 2 years of follow-up at 3 hospitals were identified (SEED alone, *n* = 775; SEED plus EBRT, *n* = 158). Of those 933 patients, 59 (6.3%) were excluded from the study (40 because of use of NHT for more than 12 months, and 19 because of use of adjuvant hormonal therapy). Of the remaining 874 patients who met the criteria for this study, 729 received SEED-BT alone, and 145 received SEED-BT plus EBRT. Based on the clinical data of the 874 patients, the 1-to-1 PSM yielded 102 pairs.

### PSM analysis

After the PSM, patient characteristics were well balanced (Table 1). Median follow-up from the initiation of radiotherapy was 95 months (range 18–153 months). In each group, 24 patients had been treated with NHT. The median duration of NHT in patients receiving it was 5.5 months (range 1–11 months) in the SEED-BT alone group and 3 months (range 1–11 months) in the SEED-BT plus EBRT group. An oral nonsteroidal anti-androgen agent alone was administered for 1 patient in the SEED-BT alone group (duration 6 months) and 7 in the SEED-BT plus EBRT group (median 3 months; range 1–11 months), respectively. An oral nonsteroidal anti-androgen agent with injection of a gonadotropin-releasing hormone agonist was administered for 20 patients in the SEED-BT alone group (median 6 months; range 1–11 months) and 17 in the SEED-BT plus EBRT group (median 3 months; range 2–9 months), respectively. An injection of a gonadotropin-releasing hormone agonist alone was administered for 3 patients in the SEED-BT alone group (median 3 months; range 3–3 months).

**Table 1:** Patients characteristics after propensity-score matching.

Variables	SEED-BT alone (n = 102)	SEED-BT plus EBRT (n = 102)	*P*
**Age at radiotherapy, median (range), years**	69 (50–81)	71 (49–79)	0.359
**PSA at diagnosis, median (range), ng/mL**	8.5 (2.6–16.7)	8.3(4.1–18.8)	0.902
**Clinical stage, number (%)**			0.322
T1c	55 (53.9)	62 (58.8)	
T2a-c	47 (46.1)	40 (41.2)	
**Gleason grade, number (%)**			0.809
1	17 (16.7)	19 (18.6)	
2	31 (30.4)	27 (26.5)	
3	54 (52.9)	56 (54.9%)	
**Positive biopsy core rate, number (%)**			0.640
< 34%	57 (55.9)	60 (58.8)	
34–67%	42 (41.2)	37 (36.3)	
> 67%	3 (2.9)	5 (4.9)	
**Unfavorable intermediate risk, number (%)**	75 (73.5)	79(77.4)	0.515
**Neoadjuvant hormonal therapy yes, number (%)**	24 (23.5)	24 (23.5)	1.000
Anti-androgen agent alone, number	1	7	
Anti-androgen agent with GnRHa, number	20	17	
GnRHa alone, number	3	0	
**Adjuvant hormonal therapy yes, number (%)**	0 (0)	0 (0)	–
**Follow-up, median (range), months**	97 (22–153)	90 (18–153)	0.046
**BED, median (range), Gy2**	200.0 (138.3–281.3)	217.1 (192.2–284.1)	< 0.001
**BED ≥ 200 Gy2, number (%)**	51 (50.0)	96 (94.1)	< 0.001
**Prostate D90, median (range), Gy**	187.7 (132.2–258.4)	124.7 (97.0–180.8)	< .0001
**Prostate V100, median (range), %**	98.9 (84.7–100)	96.7 (87.7–99.6)	< 0.001
**Prostate V150, median (range), %**	70.7 (40.1–97.2)	64.9 (39.2–87.5)	< 0.001

Prostate D90 indicates minimal dose received by 90% of prostate gland at 1 month on SEED-BT. Prostate V100 and V150 indicates percentage of prostate gland volume receiving 100% and 150% of the prescribed dose, respectively, at 1 month on SEED-BT.

BED, biochemical effective dose; EBRT, external beam radiotherapy; GnRHa, gonadotropin-releasing hormone agonist; PSA, prostate specific antigen; SEED-BT, seed brachytherapy.

**Figure 2:**
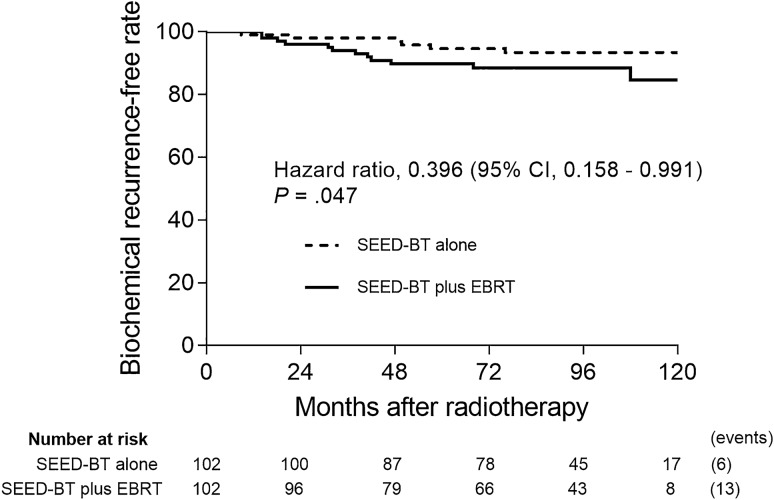
Kaplan–Meier estimates of the biochemical recurrence-free rate for patients treated with seed brachytherapy (SEED-BT) alone or with SEED-BT plus external-beam radiotherapy (EBRT), adjusted. CI = confidence interval.

**Figure 3:**
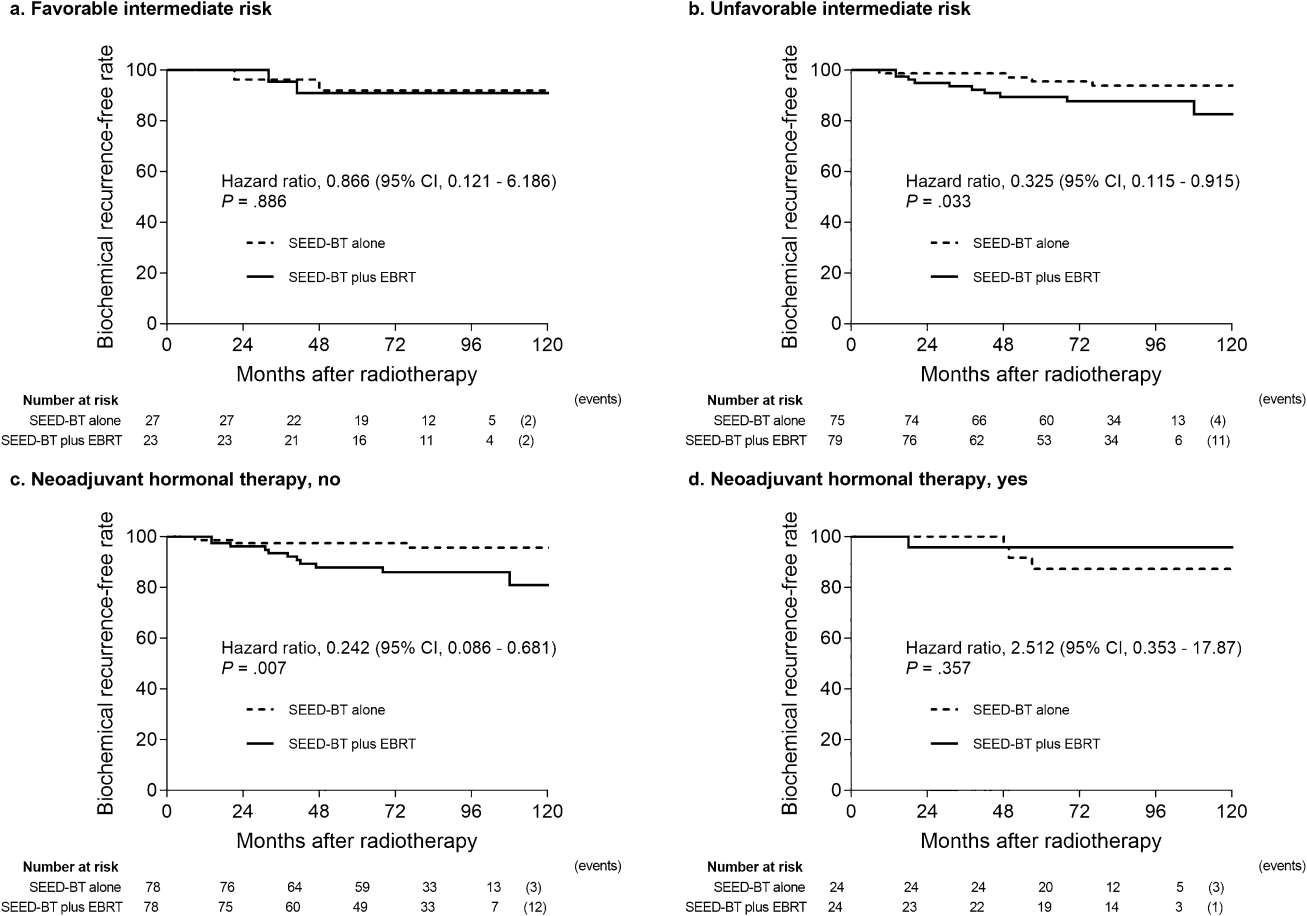
Subgroup analysis of the biochemical recurrence-free rate after propensity score-matching for patients treated with seed brachytherapy (SEED-BT) alone and with SEED-BT plus external-beam radiotherapy (EBRT). (**a**) Favorable intermediate risk subgroup. (**b**) Unfavorable intermediate risk subgroup. (**c**) Subgroup that received no concomitant neoadjuvant hormonal therapy. (**d**) Subgroup that received concomitant neoadjuvant hormonal therapy. CI = confidence interval.

The 8-year bRFR was significantly better with SEED-BT alone than with SEED-BT plus EBRT (93.3% vs. 88.4%; hazard ratio [HR] 0.396; 95% confidence interval [CI] 0.158 to 0.991; *p* = 0.047; Fig. 2). Figure 3 shows the subgroup analysis for biochemical recurrence. The 8-year bRFR was not significantly different for the favorable intermediate-risk subgroups in the SEED-BT alone and SEED-BT plus EBRT groups (91.9% vs. 90.0%; HR 0.866; 95% CI 0.121–6.186; *p* = 0.886; Fig. 3a). For the unfavorable intermediate-risk subgroups, the 8-year bRFR was significantly better with SEED-BT alone than with SEED-BT plus EBRT (93.9% vs. 87.7%; HR 0.325; 95% CI 0.115–0.915; *p* = 0.033; Fig. 3b) and with no concomitant NHT (95.6% vs. 86.0%; HR 0.242; 95% CI 0.086–0.681; *p* = 0.007; Fig. 3c). The 8-year bRFR was not significantly different for the subgroups of patients receiving concomitant NHT in the SEED-BT alone and SEED-BT plus EBRT groups (87.3% vs. 95.8%; HR 2.512; 95% CI 0.353–17.87; *p* = 0.357; Fig. 3d).

After patients either a biochemical or a clinical recurrence, 4 patients treated with SEED-BT alone and 8 treated with SEED-BT plus EBRT started salvage hormonal therapy. The 8-year salvage hormonal therapy-free rate was not significantly different for those treated with SEED-BT alone and those treated with SEED-BT plus EBRT (95.7% vs. 91.2%; HR 0.485; 95% CI 0.156–1.507; *p* = 0.211). Regional or distant metastases developed in 1 patient treated with SEED-BT alone and 6 treated with SEED-BT plus EBRT. The 8-year metastasis-free survival rate was better with SEED-BT alone than with SEED-BT plus EBRT (99.0% vs. 94.4%; HR 0.175; 95% CI 0.038–0.808; *p* = 0.025).

**Figure 4:**
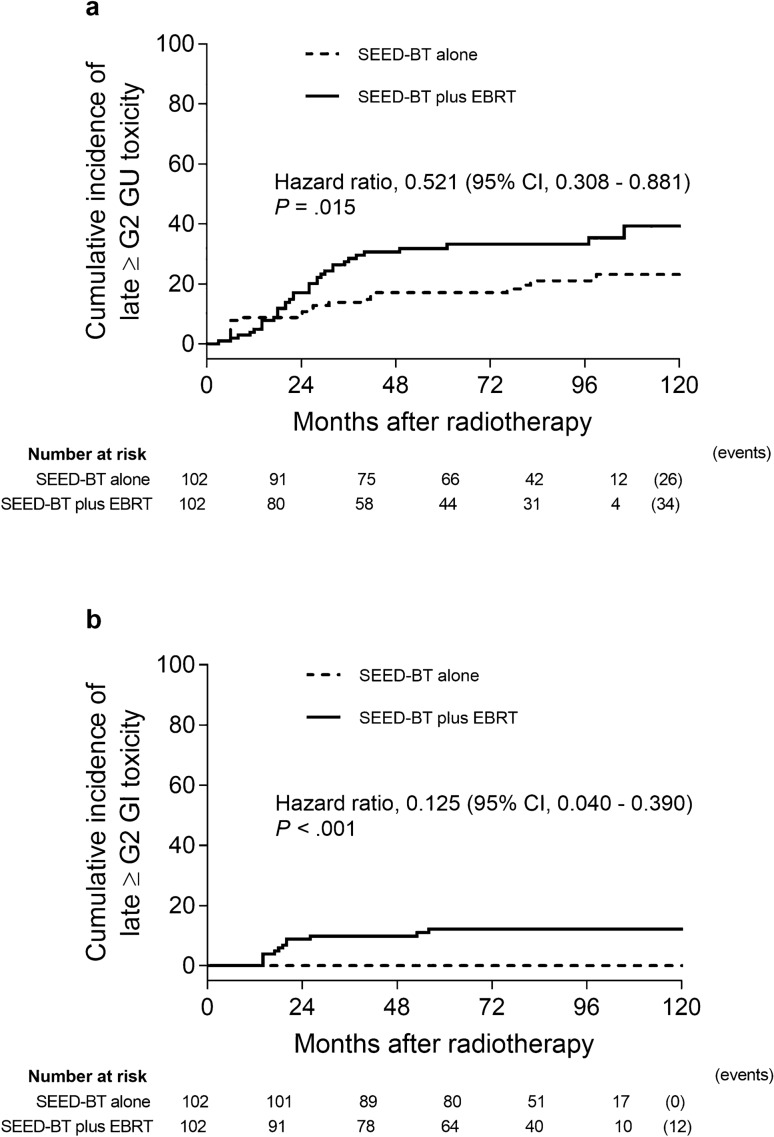
Kaplan–Meier estimates of grade 2 (G2) or greater late toxicities in patients treated with seed brachytherapy (SEED-BT) alone or with SEED-BT plus external-beam radiotherapy (EBRT), adjusted. (**a**) Genitourinary (GU) toxicities. (**b**) Gastrointestinal (GI) toxicities. CI = confidence interval.

The incidence of acute grade 2 GU toxicity was 22.5% in the SEED-BT alone group and 55.8% in the SEED-BT plus EBRT group. The incidence of acute grade 2 GI toxicity was 0% in patients treated with SEED-BT alone and 0.9% in those treated with SEED-BT plus EBRT. The cumulative incidence of grade 2 or greater late GU toxicities at 8 years was 21.0% in patients treated with SEED-BT alone and 33.2% in those treated with SEED-BT plus EBRT (Fig. 4a). Significantly fewer grade 2 or greater late GU toxicities occurred in patients treated with SEED-BT alone than in those treated with SEED-BT plus EBRT (HR 0.521; 95% CI 0.308–0.881; *p* = 0.015). Similarly, significantly fewer grade 2 or greater late GI toxicities occurred in patients treated with SEED-BT alone than in those treated with SEED-BT plus EBRT (0% vs. 12.2%; HR 0.125; 95% CI 0.040–0.390; *p* < 0.001; Fig. 4b).

The cumulative incidence of grade 3 late GU toxicities at 8 years was not significantly different in the SEED-BT alone and SEED-BT plus EBRT groups (0.9% vs. 0.0%; HR 7.142; 95% CI 0.446–114.3; *p* = 0.164). Of 102 patients treated with SEED-BT alone, 2 developed grade 3 late GU toxicity (urethral stricture and urinary retention). No patient treated with SEED-BT plus EBRT experienced a grade 3 late GU toxicity. The rate of grade 3 late GI toxicity was not significantly different at 8 years in the SEED-BT alone and SEED-BT plus EBRT groups (0.0% vs. 3.1%; HR 0.132; 95% CI 0.013–1.272; *p* = 0.079). Of 102 patients treated with SEED-BT plus EBRT, 3 developed grade 3 late GI toxicity (radiation colitis, rectal ulcer, and rectal hemorrhage). No patient treated with SEED-BT alone experienced a grade 3 late GI toxicity. No patient experienced a grade 4 or greater late GU and GI toxicity during the follow-up period.

## Discussion

We restricted our evaluation to patients diagnosed with intermediate-risk prostate cancer, and we used PSM to compare oncologic outcomes and radiation-induced toxicities between those treated with SEED-BT alone and those treated with SEED-BT plus EBRT. Kaplan–Meier curves for bRFR showed that treatment with SEED-BT alone was significantly better than treatment with SEED-BT plus EBRT. Compared with patients treated with SEED-BT plus EBRT, those treated with SEED-BT alone experienced significantly fewer grade 2 or greater late GU and GI toxicities. Those results imply that, for intermediate-risk prostate cancer, treatment with SEED-BT alone, compared with SEED-BT plus EBRT, might be a better disease-management plan.

Prestidge et al*.* provided the initial report of a phase 3 prospective randomized study (RTOG 0232) comparing treatment using EBRT followed by SEED-BT with treatment using SEED-BT alone for patients with intermediate-risk prostate cancer^19^. At the interim analysis, 5-year progression-free survival was 85% in the EBRT plus SEED-BT arm and 86% in the SEED-BT alone arm. Their report confirmed that, compared with treatment using SEED-BT alone, the addition of EBRT did not result in a superior 5-year progression-free survival rate (median follow-up was 6.7 years). Grimm et al*.* reported an analysis of bRFR in the treatment of intermediate-risk prostate cancer. They suggested that combination brachytherapy and EBRT appears to be equivalent to brachytherapy alone^2^. Those previous studies accord with the favorable results in terms of biochemical control that we observed for SEED-BT alone compared with SEED-BT plus EBRT.

BED equations show that, of the various radiotherapy approaches, the combination of SEED-BT and EBRT delivers the higher dose to the prostate^16^. And, in fact, median BED was higher in the SEED-BT plus EBRT group than in the SEED-BT alone group in the present study (217.1 Gy2 vs. 200 Gy2, *p* < 0.001). Additionally, a BED of 200 Gy2 or greater was reached for more patients in the SEED-BT plus EBRT group (96.1%) than for patients in the SEED-BT alone group (50%). A higher BED and more frequent achievement of a BED of 200 Gy2 or greater usually result in better oncologic outcomes^20,21^; however, in the present study, the higher BED of SEED-BT plus EBRT was not reflected in a better bRFR. The higher BED in the SEED-BT plus EBRT group seemed to increase late GU and GI toxicities. Those results may indicate that reaching the BED level of ≥ 200 Gy2 using the combination of SEED-BT plus EBRT may not be as effective in improving bRFR when compared with higher quality implant dose delivery using SEED-BT alone. We believe that reaching the higher level of prostate D90 using SEED-BT alone with controlling urethral and rectal doses is important to improve bRFR rather than reaching the higher level of BED with the additional EBRT. Kao et al*.* suggested that high prostate D90 of ≥ 180 Gy resulted in excellent biochemical control with the tolerable toxicities in patients treated with SEED-BT alone^22^. The high level of prostate V150 in the SEED-BT alone group could also affect the better bRFR in the present study. The brachytherapy can make high dose areas in the vicinity of radioactive sources, whereas there are no such high dose areas in the EBRT. While the high dose areas have some biological advantages for the cancer control^23^, the BED equations for SEED-BT does not consider the high dose areas such as prostate V150^16^. The BED equations, in which only the prostate D90 is included in the calculating formula as dose volume histogram parameters, may underestimate the therapeutic effect of SEED-BT.

The techniques for SEED-BT have been refined over time, such as changing from the preplanning method to the intraoperative planning method, and from uniform loading (the Initial Seattle approach) to modified peripheral loading. The newer techniques using SEED-BT alone deliver higher doses to the prostate by controlling the dose to organs at risk and might contribute to the improvement in bRFR and the toxicity profile in patients receiving this treatment.

After PSM, more than 70% of the patients in this study had disease classified as unfavorable intermediate-risk. In that subgroup of patients with unfavorable intermediate-risk disease, the bRFR was better with SEED-BT alone than with SEED-BT plus EBRT. Thus, treatment with SEED-BT alone appears to have the potential to improve bRFR even for patients with disease classified as unfavorable intermediate-risk. In addition, the 8-year bRFR was significantly better with SEED-BT alone than with SEED-BT plus EBRT in the subgroup receiving no concomitant NHT (95.6% vs. 86.0%). SEED-BT alone, without NHT, might provide a sufficiently high biochemical control rate in the treatment of intermediate-risk prostate cancer. King et al*.* investigated the relative benefit of supplemental therapies including androgen deprivation therapy and EBRT for unfavorable intermediate risk. Supplemental EBRT (adjust HR 2.66; 95% CI 1.12–6.35), androgen deprivation therapy (adjust HR 0.96; 95% CI 0.38–2.43), or both (adjust HR 1.46; 95% CI 0.42–5.01) were not associated with improved prostate cancer-specific mortality^24^. The others reported that the addition of androgen deprivation therapy demonstrated no benefit on bRFR or metastasis-free rate in unfavorable intermediate-risk treated with SEED-BT alone^25,26^. Those results supported that SEED-BT alone without NHT could be one of the best treatment options for unfavorable intermediate-risk. Although no consensus that supports the superiority of SEED-BT plus EBRT over SEED-BT alone has developed, the recent U.S. National Comprehensive Cancer Network guidelines (2022, version 1) recommend combined SEED-BT and EBRT, but not SEED-BT alone, in patients with unfavorable intermediate risk disease. Long-term clinical outcomes from the RTOG 0232 study would answer the question of whether SEED-BT plus EBRT is superior to SEED-BT alone in the management of intermediate-risk prostate cancer. However, compared with patients having favorable intermediate-risk disease, those with unfavorable intermediate-risk disease were not likely to meet the inclusion criteria for the RTOG 0232 study. Another prospective randomized trial open to patients with unfavorable intermediate-risk disease would be needed to answer the question.

Yorozu et al*.* reported the 7-year actuarial risk of grade 2 or greater GU and GI toxicity developing in 1313 patients undergoing SEED-BT, of whom, 48% received EBRT^27^. Compared with patients treated with SEED-BT alone, those treated with SEED-BT plus EBRT experienced a significantly higher incidence of grade 2 or greater GU toxicity (24.3% vs. 18.6%, *p* = 0.016). In the same report, patients treated with SEED-BT plus EBRT also experienced a significantly higher incidence of grade 2 or greater GI toxicity (12.6% vs. 1.9%, *p* < 0.001). In that report and in the present study, the slopes on the Kaplan–Meier curves for the incidence of grade 2 or greater GU and GI toxicity were similar. The RTOG 0232 trial showed that, after treatment with SEED-BT plus EBRT and with SEED-BT alone, overall rates of grade 3 or greater late GU toxicity were 7% and 3% respectively, and rates of grade 3 or greater GI toxicity were 3% and 2%^19^. Yorozu et al*.* also reported that, after treatment with SEED-BT plus EBRT and with SEED-BT alone the 7-year actuarial probabilities of grade 3 toxicity were 2% for GU toxicity and 0.7% for GI toxicity^27^. Although the incidences of grade 3 or greater toxicity in our cohort did not depart from the incidences in previous reports, the trends in our cohort differed somewhat, in that grade 3 late GU toxicity was seen only in patients treated with SEED-BT alone and grade 3 late GI toxicity was seen only in patients treated with SEED-BT plus EBRT.

Several potential limitations of the present study must be considered in addition to its retrospective nature. First, our results might not be generalizable to other institutions because they represent the pooled experience of 3 tertiary centers. The 3 institutions had quite different approaches to managing patients with intermediate-risk disease, such as the sequence in which EBRT was added and the inclusion criteria for treatments. Those varying approaches and qualities of brachytherapy could potentially have affected our outcome measures. In addition, the use of bRFR might be weak as a clinically meaningful primary endpoint in early-stage prostate cancer. Overall survival and prostate-cancer-specific survival are more relevant than biochemical recurrence. Survival data from multiple centers involving more patients with longer follow-up periods will clarify the superiority or inferiority of these treatments. Second, we did not evaluate patient-reported outcomes or erectile dysfunction after treatment with SEED-BT alone or SEED-BT plus EBRT. Third, we could not define the optimal dose for ^125^I prostate implants when considering the balance between biochemical recurrence and radiation-induced toxicities^28^. Fourth, we included the presence or absence of NHT for calculating the propensity score, but because of the limited number of patients receiving NHT, we did not include the durations or types. The varying types and durations of NHT might affect the bRFR after radiotherapy^29^. Thus, evaluating the effect of NHT on biochemical recurrence was not adequate in the present study.

## Conclusions

The results of the present study imply that, compared with SEED-BT plus EBRT, SEED-BT alone might be a better disease-management plan for patients with intermediate-risk prostate cancer. Our resulting hypothesis is that adding EBRT to SEED-BT does not result in superior oncologic outcomes in intermediate-risk prostate cancer regardless of favorable and unfavorable characteristics. SEED-BT alone without NHT might provide a sufficiently high biochemical control rate in the treatment of intermediate-risk prostate cancer. A prospective evaluation of the role of SEED-BT alone is required to address that hypothesis and the current controversies with respect to the guidelines for patients with unfavorable intermediate-risk disease.

## Data Availability

The datasets generated during and/or analysed during the current study are not publicly available because the protocol did not include a data sharing plan but are available from the corresponding author on reasonable request.
